# A Hybrid Care Intervention for High-Risk Patients With Chronic Obstructive Respiratory Disorders: Mixed Methods Co-Design Study

**DOI:** 10.2196/96600

**Published:** 2026-08-18

**Authors:** Alba Gómez-López, Núria Sánchez-Ruano, Marta Sorribes, Rubèn González-Colom, Marina Paredes, Jin-Seok You, María José Gordillo, Emili Vela, Gerard Carot-Sans, Alba Jiménez, Xabier Michelena, Ramon Farré, Josep Roca, Isaac Cano, Alicia Aguado, Jose Fermoso, Néstor Soler, Ebymar Arismendi

**Affiliations:** 1 Department of Pneumology, Hospital Clínic de Barcelona, C/Rosello 149-153, Barcelona, Catalonia, Spain, 34 932275400; 2 Physiopathological Mechanisms of Respiratory Diseases, Fundació de Recerca Clínic Barcelona-Institut d’Investigacions Biomèdiques August Pi i Sunyer, C/ Rosselló, 149-153, Barcelona, Catalonia, 08036, Spain; 3 School of Medicine and Health Sciences, Universitat de Barcelona, C/ Casanova, 143, Barcelona, Catalonia, Spain; 4 Centro de Investigación Biomédica en Red de Enfermedades Respiratorias (CIBERES), Instituto de Salud Carlos III, Av. Monforte de Lemos, 3-5, Madrid, Madrid, Spain; 5 CAP Comte Borrell, Consorci d'Atenció Primària de Salut de Barcelona Esquerra (CAPSBE), Barcelona, Catalonia, Spain; 6 CAP Numancia, Institut Català de la Salut, Barcelona, Catalonia, Spain; 7 Digitalization for the Sustainability of the Healthcare System (DS3), Barcelona, Catalonia, Spain; 8 Catalan Health Service, Government of Catalonia, Barcelona, Catalonia, Spain; 9 Fundació TIC Salut i Social, Barcelona, Catalonia, Spain; 10 Clinical Informatics Service, Hospital Clínic de Barcelona, Barcelona, Catalonia, Spain; 11 CARTIF Technology Center, Valladolid, Castille and León, Spain

**Keywords:** chronic obstructive pulmonary disease, community-based research, co-design, nurse-led care, severe asthma, patient-centered care

## Abstract

**Background:**

Community-based management of exacerbations in high-risk patients with chronic obstructive respiratory diagnoses remains a major challenge. Hybrid care interventions, combining digital support with in-person, patient-centered care, have shown efficacy to reduce unplanned hospitalizations in controlled trials. However, an efficacy-effectiveness gap remains, indicating the complexities of its deployment and sustainable adoption in real-world scenarios.

**Objective:**

This study aimed to co-design the core components of a hybrid care intervention for the preventive management of exacerbations in high-risk patients with chronic obstructive respiratory conditions, generating insights to guide sustainable adoption in routine clinical practice.

**Methods:**

Four plan-do-study-act (PDSA) co-design cycles were conducted, using a convergent mixed methods approach, during the 2-year follow-up (2024‐2025) of a cohort of 205 high-risk patients pertaining to two different clinical programs: (1) the community-based program included multimorbid patients with chronic obstructive pulmonary disease (COPD), asthma, or bronchiectasis from the Integrated Health District of Barcelona-Esquerra (AISBE, 520 k citizens) and (2) the Severe Asthma Program included patients with severe asthma. In all cases, patients were managed following the corresponding disease-specific consensus guidelines. The specific aims of each PDSA cycle were: PDSA-1, patients’ profiling and applicability of technological tools; PDSA-2, definition of clinical aspects of the hybrid care intervention and refinement of technology; PDSA-3, evaluation of a consolidated version of the hybrid care intervention; and PDSA-4, final refinements.

**Results:**

At the end of PDSA-3 (August 2025), the operationalization of the three core components of hybrid care—(1) nurse-led in-person care, (2) personalization of the intervention, and (3) advanced digital support—was achieved. The main study outcome was established through consensus among all key stakeholders on two aspects: (1) applicability of the hybrid care intervention for management of these patients in clinically stable conditions and during exacerbations in the real-world setting and (2) a well-defined strategy for its short-term deployment and sustainable site adoption.

**Conclusions:**

The intervention rollout requires emphasis on (1) alignment with local care pathways and information systems, (2) clear role definition and escalation procedures across care tiers, and (3) adaptation of the digital layer to patients’ capabilities, including pragmatic support for those with limited digital literacy. The co-design process enabled the operationalization of a hybrid care intervention integrating nurse-led management, personalization of care, and advanced digital support. Stakeholders reached consensus regarding its applicability and implementation strategy. Future real-world implementation studies are needed to evaluate its effects on clinical outcomes, health care use, patient experience, and health care value generation.

## Introduction

Patients with chronic obstructive pulmonary disorders frequently experience unplanned hospitalizations, partly attributable to suboptimal management of multifactorial acute episodes at the community level [1-4]. These events negatively affect health-related quality of life, worsen prognosis, and disproportionately consume health care resources [5]. A substantial proportion of these admissions are considered potentially avoidable, underscoring the urgent need for effective preventive strategies in community-based care [6]. Although consensus guidelines are available for inpatient management of severe exacerbations [7], effective strategies to address acute events in the community setting remain a pressing priority [5-8].

Growing evidence supports the efficacy of early detection and personalized, community-based care in reducing hospitalization rates, improving clinical outcomes, and enhancing quality of life, while simultaneously alleviating pressure on health care systems, addressing a critical unmet need in global health policy [9-11]. Hybrid care interventions adopting a patient-centered approach, combining in-person clinical care with digital tools for communication, monitoring, and decision support, represent a logical extension of these strategies and have the potential to facilitate and enhance the early management of acute episodes across community settings [11-13]. Nevertheless, a persistent gap remains between the outcomes reported in randomized controlled trials (RCTs) and the real-world effectiveness of these approaches [13].

This gap appears to be driven by persistent challenges related to intervention design, personalization, and implementation [14]. Patients eligible for community-based management are frequently older adults, multimorbid, and socially heterogeneous, with substantial variability in functional status and digital literacy [14,15]. At the same time, health care organizations are required to integrate new care modalities within established workflows, professional roles, and information systems [14,15]. Under these circumstances, hybrid care interventions may fail to be adopted or sustained, not because of insufficient effectiveness, but because their components are not adequately operationalized, tailored, or aligned with routine clinical practice. From this perspective, greater attention is needed to the systematic co-design and operational refinement of hybrid care interventions that are innovative, feasible, and adaptable to real-world clinical settings.

In this study, we report a 2-year follow-up study of a cohort of 205 patients with chronic obstructive respiratory disorders at high risk for exacerbations, in whom a hybrid care intervention was piloted [16]. The study protocol [17] included a co-design process, involving all stakeholders, through 4 plan-do-study-act (PDSA) sequential cycles [17,18], each lasting 6 months. Our objective was to operationalize the core components of hybrid care, identify patient profiles relevant for personalization, and generate implementation-oriented insights to inform future deployment and evaluation in routine clinical practice.

## Methods

### Study Design

The study was conducted in the catchment area of the Hospital Clinic de Barcelona (HCB), within the Integrated Health District of Barcelona-Esquerra (AISBE, approximately 520 k citizens). The current research adopted a convergent mixed methods design [19-21], integrating quantitative and qualitative data to iteratively refine the hybrid care intervention. Quantitative data were used to characterize the study population, support patient profiling, and test the technology, whereas qualitative data provided contextual and experiential insights to inform the co-design process.

Table 1 describes the main characteristics of the study design for the qualitative component of the research. The information for qualitative analyses relied on three main sources: (1) longitudinal experiential data collected by the nurse case manager during routine interactions with patients and caregivers (including chat-based communication and follow-up contacts), (2) continuous feedback from the different supporting teams described in Table 1 that was compiled and synthesized in the weekly sessions carried out by the core research team across the PDSA cycles, and (3) a structured focus group conducted at the end of PDSA-3 to consolidate the strategy for deployment and sustainable adoption of the hybrid care approach in real-life clinical care. Qualitative data were synthesized iteratively by the research team and integrated with quantitative findings to inform decision-making and refinement of the hybrid care intervention components.

Each PDSA cycle simultaneously addressed the following four components, briefly described in the table: (1) structure of the nurse-led hybrid care intervention, (2) patients’ risk profiling and personalization of care, (3) advanced digital support, and (4) household indoor air quality (IAQ) assessment using low-cost sensors (LCS). Details on integration between outcomes from qualitative analyses and quantitative assessments for the 4 blocks reported in Table 1, within the relevant PDSA cycles, are described in detail in the results section.

### Patient Recruitment

The study cohort included patients visited in the context of two distinct clinical programs: (1) a community-based program for patients with chronic obstructive respiratory disorders, and (2) patients with severe asthma [2].

The first step for the enrollment of patients in the community-based program was elaborating the dataset of potential candidates using registry information from the Catalan Health Surveillance System (CHSS) [22], allowing the identification of the corresponding primary care physicians. The primary care physicians’ engagement in the study facilitated initial contact with the patients, exploring their willingness to participate and keeping them within their health care team during the follow-up period. An initial phone visit by the nurse case manager, on behalf of the primary care team, was used to introduce the project and set the first face-to-face visit to the patient’s home.

Inclusion and exclusion criteria for the community-based program were: (1) adults below 85 years, (2) residents in AISBE, and (3) with a diagnosis of chronic obstructive pulmonary disease (COPD), asthma, or bronchiectasis, and showing high multimorbidity burden [23]. Patients with dementia or those who were unable to independently perform activities of daily life were excluded from the study. Also, patients already enrolled in other chronic care programs such as home care support for advanced chronic complex patients, or palliative care, were not included in the study cohort.

Approximately 20% of the cohort was targeted for inclusion of patients with severe asthma [23] recruited from the outpatient Severe Asthma Unit of the Pulmonology Department of Hospital Clinic de Barcelona (HCB) following similar steps. Further details on the inclusion and exclusion criteria were listed in the reported study protocol [17].

### The Co-Design Process

As described in Table 1, the core research team (AGL, RGC, ICF, and JRT) implemented the 2-year (2024‐2025) co-design process to gain clinical applicability of the hybrid care intervention. To achieve this, 4 sequential PDSA co-design cycles, each lasting 6 months, were conducted by the research group with active continuous contributions of: (1) the patients and caregivers from the study cohort, (2) four clinical teams of health professionals: 2 primary care centers from AISBE, and 2 teams of respiratory specialists from HCB, one of them providing support to the community-based program (AISBE) and the second running the severe asthma unit at HCB, (3) two external technical support teams, and (4) stakeholders from the Digitalization for the Sustainability of the Healthcare System (DS3 group) pertaining to the single regional public payer in Catalonia (CatSalut), as described in detail in Appendix 1 in Multimedia Appendix 1.

The first PDSA cycle (PDSA-1, January to June 2024) had two main objectives: (1) to address patients’ profiling, and (2) to assess feasibility, beyond clinical research context, of the 4 components indicated in Table 1, in particular the technological tools used in the follow-up of the cohort: digital platform [12], heart rate variability (HRV) monitoring, respiratory oscillometry testing, and household IAQ monitoring with LCS.

PDSA-2, from July to December 2024, addressed the following aspects: (1) completion of patients’ profiling, (2) definition of clinical aspects of the hybrid care intervention with an integrated care approach, (3) elaboration of strategies for patients’ risk assessment and personalization of care, and (4) refinement of different aspects of the technological support.

The third PDSA cycle (PDSA-3, from January to September 2025) evaluated a consolidated version of the hybrid care intervention in the study cohort, introducing some refinements.

The patients’ and caregivers’ experience and outcomes regarding the characteristics of the hybrid care intervention were collected by the nurse case manager during the follow-up of the study cohort. The outputs from the co-design cycles were collectively evaluated by a dedicated focus group session with 14 leading professionals, mixing clinical staff (n=9), IT developers (n=1), data scientists (n=1), and managers (n=3) to consolidate findings and reach a final consensus on the structure of the integrated hybrid care intervention and the implementation strategy, aiming at fostering sustainable adoption in routine practice.

**Table 1.** Study design: qualitative component.

	Content to be defined and objectives
Co-design blocks	
Block 1 (B1): nurse-led hybrid care intervention	Definition of roles of the nurse in the management of patients’ care pathways both clinical stability and exacerbations. Specification of nurse profiles: community-based case manager versus advanced practice nurse. Characterization of interactions with the reference physician (primary care or specialist care), other health care and social support professionals. Management of technological support provided to patients.
Block 2 (B2): personalization of care	Identification of critical dimensions to be considered for personalization of patients’ care pathways: disease severity, comorbidities, functional impairment, socioeconomic vulnerability, digital literacy. Strategies for development, evaluation, and sustainable implementation of clinical decision-support models.
Block 3 (B3): digital support	Refinement of the digital platform (Health Circuit). Evaluation of the applicability and clinical utility of heart rate variability (HRV)^a^ and respiratory oscillometry.
Block 4 (B4): home IAQ^b^ monitoring	Explore applicability and potential clinical role of household IAQ monitoring using LCS^c^.
Participants *(*see Patient’s Recruitment for details)	Core research team (n=4), patients (n=205), clinical teams (n=4), technical teams (n=2), DS3^d^
Prioritization within co-design blocks for each PDSA^e^ cycle (see Study Design and The Co-Design Process for details)	
PDSA-1	Assessment of the feasibility of key components of each of the 4 blocks, as defined in [17]. Identification of weak points and design of contingency plans.
PDSA-2	B1: Granularities of the intervention defined. B2: Assessment of the impact on hybrid care of the five critical dimensions: disease severity, comorbidities, functional impairment, socioeconomic vulnerability, digital literacy. B3: Refinement of the digital platform and the two target tests: Oscillometry and HRV. B4: Maturity of household IAQ monitoring applicability.
PDSA-3	B1: Consensus on the adoption of hybrid care models in routine clinical practice beyond the study protocol. B2: Proposals for stratification of patients in the 2 clinical programs. B3: Further refinement of the digital platform; final decisions regarding HRV monitoring; and recommendations for the use of oscillometry in stable patients and during exacerbations.
PDSA-4	Complete refinements and plan real-life deployment beyond the report.
Logistics & sources of information (see Patient Profiling for details)	
Patients-nurse interactions	Data sources: (1) questionnaires, (2) patient-nurse chat logs, and (3) information collected during routine contacts. Data were weekly reported to the core research team.
Core research team and supporting teams	Core research team weekly meetings: (1) to integrate info from patients and supporting teams, and (2) to modulate priorities within each PDSA phase.
Focus group session	Conducted at the end of PDSA-3 to consolidate the current report

a HRV: heart rate variability.

b IAQ: indoor air quality.

c LCS: low-cost sensor.

d DS3: Digital Support for Sustainability of the Catalan Health System, research and innovation team from the regional single public payer (CatSalut).

e PDSA: plan-do-study-act.

### Patient Profiling

Contextual data were collected to characterize the study population across multiple dimensions, including sociodemographic, clinical, and functional characteristics, unhealthy lifestyle habits, multimorbidity burden [23,24], disabilities, social frailty, use of health care resources, and digital literacy. Adjusted morbidity groups (AMG) scoring reflects both the number and complexity of morbidity. The index is extensively used in Spain and other European regions, as reported in detail in [25]. Such comprehensive assessment of the study cohort aimed at identifying the candidates’ profiles for a personalized hybrid care intervention.

All data for patient profiling were gathered from 3 sources. At baseline, the nurse case manager instructed the patients, administered the different questionnaires, and performed lung function testing through 2 home visits of 1 hour duration each. Clinical information and therapies were gathered from the hospital care electronic records, with access to primary care information. A second, midterm assessment of the cohort was carried out, and a third home visit was planned at the end of the follow-up, as reported in [17]. We used the CHSS [22] to extract historical data on resource use, multimorbidity [23,24], and sociodemographic data. The CHSS centralizes data from primary and specialized care across the entire health care system.

Clinical characterization: at baseline, the patients were clinically characterized using a comprehensive set of variables, including sociodemographic data, primary respiratory diagnosis, comorbidities, lung function using forced spirometry and oscillometry, and health care resource use in the 12 months preceding enrollment (primary care visits, emergency department visits, hospital admissions, and total health care expenditure). Functional capacity was assessed using standardized patient-reported outcomes evaluating physical and mental health as well as respiratory symptoms and overall health status [26-34]. Additional variables included lifestyle risk factors (smoking, alcohol, physical activity, passive smoking), as well as housing characteristics and living conditions described in [17].

Digital literacy profiling: digital literacy was assessed with a purpose-designed questionnaire comprising different items evaluating knowledge, usage, and confidence in digital skills relevant to the hybrid care model. Results were compared with a parallel classification into 5 levels of digital skills (none, low, medium with no improvement capacity, medium with improvement capacity, high), based on the engagement and support needs observed by the nurse.

Respiratory diagnoses profiling: to ensure diagnostic accuracy, the single respiratory diagnosis validated by the research team examining the patients’ electronic health care records was compared with the respiratory diagnoses obtained from the CHSS, including diagnostic information across all health care tiers.

### Statistical Considerations

Continuous variables are reported as mean (SD) or median (IQR, defined as the 25th and 75th percentiles), as appropriate; categorical variables are presented as n (%). Between-group comparisons for continuous data used the student’s *t* test when distributional assumptions were met and the Wilcoxon rank-sum test otherwise. Categorical data were compared with the Fisher exact test. All tests were 2-sided, and *P*<.05 was considered statistically significant. All the analyses have been conducted using R (version 4.1.1; R Core Team) .

### Ethical Considerations

Ethical approval for the process evaluation was granted by the Ethical Committee for Human Research at the HCB on June 29, 2023 (HCB/2023/0126) and registered at ClinicalTrials.gov (NCT06421402). The patients participating in the research were required to provide and sign a written informed consent indicating the purpose of the study, the nature, use, and management of their data. Participants did not receive any compensation for their participation.

## Results

### Overview

The Results section is organized into 2 main subsections. Section A presents the characteristics of the study cohort involved in the co-design process. Section B summarizes the key findings and lessons learned from the first 3 PDSA cycles, which informed the iterative development of the hybrid care intervention for routine clinical practice, and describes the refinements identified and incorporated during the fourth PDSA cycle to optimize the final intervention.

### Characterization of the Study Cohort

**Figure 1.**
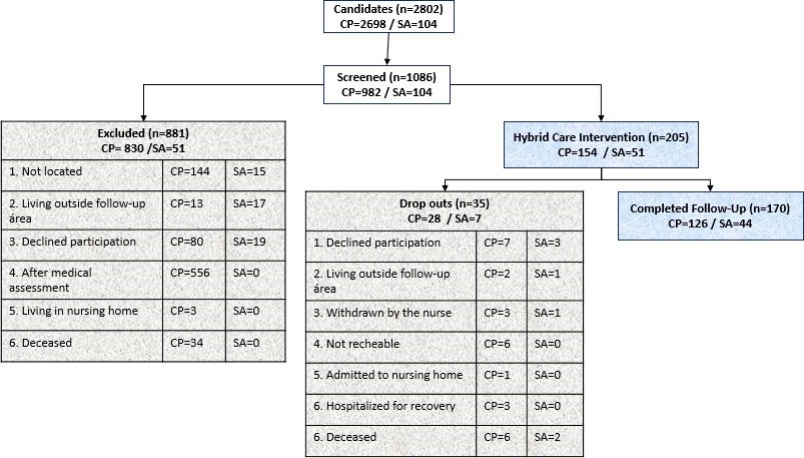
Study flow diagram. CP: community-based program in AISBE (Integrated Health District of Barcelona-Esquerra); SA: Severe Asthma unit program.

During the recruitment period, 205 patients were enrolled in the study: 152 (74%) from the community program (AISBE) and 53 (26%) from the severe asthma group (Figure 1). Of the 205 patients enrolled, 22 (10.7%) were excluded from analysis (19 withdrawals, 3 deaths), resulting in an analytic cohort of 183 participants.

Table 2 summarizes baseline clinical characteristics for (1) the overall analytic cohort (n=183), (2) community-managed patients in the AISBE program (n=135; 74%), and (3) patients followed at HCB’s Severe Asthma Unit (n=48; 26%).

Compared with patients from the severe asthma unit, the community-managed patients with chronic obstructive respiratory diseases were older (72.4 vs 57.4 y; *P*<.001), more often male (51.9% [70/183] vs 18.8% [9/183]; *P*<.001), and had a higher comorbidity burden (adjusted morbidity groups [AMG] 83.8 vs 63.7; *P*=.001). Lung function indicated greater airflow limitation in AISBE (forced expiratory volume in 1 second (FEV_1_) % predicted 57.3% [135/183] vs 82.6% [48/183]; *P*<.001; FEV₁/FVC 48.5 vs 83.4; *P*=.01). No differences in the hospitalization rate during the previous year were observed between the 2 groups (all-cause hospitalization 35.8% [48/183] vs 33.3% [16/183], *P*=.86; unplanned 27.6% [37/183] vs 22.9% [11/183], *P*=.57). Health care use over the prior year was broadly similar between groups. We found no significant differences in health care expenditure per patient between the AISBE group (€5133 [US $5544]) and the severe asthma group (€8101 [US $8749]) (*P*=.11). US $ values were calculated using the average 2024 exchange rate of €1=US $1.08.

**Table 2.** Baseline characteristics of the study groups (n=183). Total health care expenditure corresponds to total expenditure per year across all health care tiers. The *P* value corresponds to difference analysis between patients recruited from the 2 programs.

Variables	All patients (n=183)	Patients followed by primary care (AISBE^a^) (n=135)	Patients followed by the severe asthma unit (n=48)	*P* value
Age, mean (SD)	68.45 (11.98)	72.38 (8.52)	57.42 (13.43)	<.001
Sex, n (%)				
Male	79 (43.17)	70 (51.85)	9 (18.75)	<.001
Female	104 (56.83)	65 (48.15)	39 (81.25)	<.001
Main respiratory diagnosis and comorbidities				
Severe asthma, n (%)	48 (26.23)	0 (0)	48 (100)	—^i^
Asthma, n (%)	15 (8.20)	15 (11.11)	0 (0)	—
COPD^b^; n (%)	97 (53.01)	97 (71.85)	0 (0)	—
Bronchiectasis, n (%)	23 (12.57)	23 (17.04)	0 (0)	—
AMG score^c^, mean (SD)	78.53 (46.27)	83.82 (45.26)	63.74 (46.32)	.001
Lung function				
Spirometry				
FEV1^d^ %, mean (SD)	63.83 (25.16)	57.28 (22.79)	82.64 (22.16)	<.001
FEV1^d^/FVC^e^^f^, mean (SD)	52.17 (16.61)	48.50 (13.10)	83.40 (6.51)	.01
Oscillometry				
R5-20 kPa·s/l ^g^, mean (SD)	0.13 (0.13)	0.13 (0.12)	0.13 (0.14)	.81
AX kPa/l ^h^, mean (SD)	2.24 (2.01)	2.42 (2.04)	1.77 (1.85)	.049
Use of health care resources during the 12 months before inclusion in the study				
Hospitalizations, n (%)	64 (35.16)	48 (35.82)	16 (33.33)	.86
Unplanned hospitalizations, n (%)	48 (26.37)	37 (27.61)	11 (22.92)	.57
Total health care expenditure per patient in 2024 €^j^, median (IQR)	5658 (2890-12,084)	5133 (2890-10,611)	8101 (3543-16,045)	.11

a AISBE: Integrated Health Care Barcelona Esquerra.

b COPD: chronic obstructive pulmonary disease.

c AMG score: adjusted morbidity groups score, indicating comorbidities and complexity [24].

d FEV1: forced expiratory volume in 1 second.

e FVC: forced vital capacity.

f FEV1/FVC: ratio of FEV1 to FVC; oscillometry.

g R5–20 kPa·s/l: difference between respiratory resistance at 5 Hz and 20 Hz.

h AX kPa/l: reactance area.

i Not available.

j the average 2024 exchange rate was €1=US $1.08.

### Disability Profile and Socioeconomic Context

The World Health Organization Disability Assessment Schedule (12-item WHODAS questionnaire) [34] reflected graded levels of disability in 6 domain-specific scores. The mean WHODAS score was 1.88 (SD 0.77, IQR 1.5‐2.4), consistent with mild disability, as expected due to the entry criteria. Within the specific disability domains, a substantial interindividual dispersion was observed. Furthermore, a significant number of patients reported socioeconomic barriers, such as limited financial resources (<16,000€/y [US $17,300/y]; 74/160, 46.3%; 23/183, 12.5% missing), or living alone (45/183, 24.6%), highlighting potential sources of inequity that could influence patients’ capacity for effective self-management. (Appendices 2 and 3 in Multimedia Appendix 1).

### The Co-Design Process of the Hybrid Care Intervention

#### Overview

**Figure 2.**
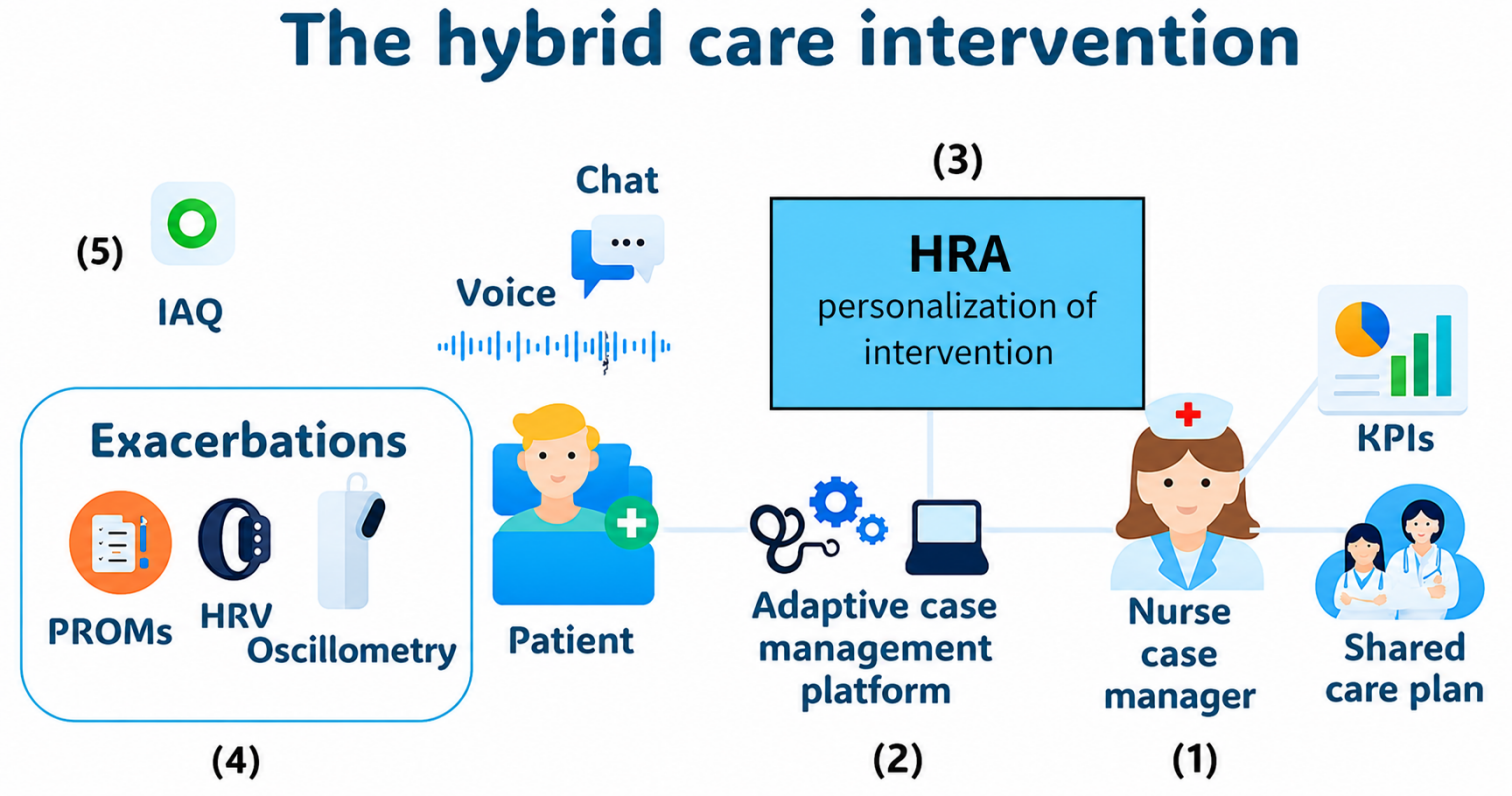
Co-design process of the hybrid care intervention: (1) roles of the nurse case manager, (2) advanced digital support using an Adaptive Case Management Platform (Health Circuit) [12], (3) personalization of the intervention through health risk assessment [35,36], (4) characterization of acute episodes using daily home-based patient’s self-administered oscillometry measurements (lung function testing) [37], heart rate variability monitoring (autonomic regulation) [38], and symptoms assessed with a Visual-Analogic Scale, and (5) assessment of household indoor air quality using low-cost sensors [39] (Appendix 1 in Multimedia Appendix 1). HRA: health risk assessment; KPI: key performance indicator; PROM: patient-reported outcomes measure.

Throughout the co-design process, qualitative feedback from patients and stakeholders played a key role in shaping the operationalization of the intervention components. At the end of PDSA-3, as described in this section, participants reached a consensus regarding operationalization of the three core elements of a patient-centered service that combines digital support and in-person care: (1) nurse-led integrated care, (2) advanced and flexible digital support, and (3) patient-based health risk assessment for personalization of the hybrid care intervention (Figure 2 and Textboxes 1–4).

Textbox 1. **Elements in a digitally enabled care continuum setting.**

**Proactive patient**: fulfilling inclusion criteria. Empowered for self-management. Accessibility to the nurse through a multimedia communication channel (app) and digitally enabled patient’s self-capture data system which includes structured and nonstructured data, including self-administered short questionnaires, and monitoring of physiological variables, such as heart rate (HR), heart rate variability (HRV), activity levels, pulse oximetry, etc., using wrist sensors

**Nurse case manager**: based in the community or at specialized clinics, depending upon the clinical program. Accessibility to the patient and to the reference physician (or other health care professionals) through a multimedia communication channel (web). Digitally enabled with the professional’s dashboard

**Reference physician**: primary care physician or specialist depending upon the clinical program. Accessibility to the nurse case manager (to other health care professionals and to the patient) through a multimedia communication channel (app).

Textbox 2. **Clinical programs.**

**Community-based AISBE (Integrated Health District of Barcelona-Esquerra) program**: targeting comorbid patients with chronic obstructive respiratory disorders and an Adjusted Morbidity Groups (AMGs) [23,24] scoring above percentile eighty in the health risk regional population pyramid (Table 1). The general practitioner serves as the reference physicianfor these patients. The interactions with other service modalities are fully aligned with the preexisting shared care agreements at health district level (520 k citizens).

**Severe Asthma Clinic program**: targeting asthma patients, stages 5 and 6, controlled in the Severe Asthma Unit at Hospital Clínic de Barcelona (HCB). One advanced care nurse could lead the program.

Textbox 3. **Management of clinically stable patients.**

- The first step is the patient’s motivational interview conducted by the nurse aimed at: (1) empowering the patient for self-management of their condition, and (2) facilitating the collaborative development of a patient’s tailored care plan, with a holistic approach, agreed upon by the patient, the nurse, and the reference physician.
- The care plan forms the basis for the patient’s periodic follow-up by the nurse case manager, with most interactions taking place through the established communication channel. In all cases, the management plan takes into consideration the diversity of patients’ profiles.
- The clinical management plan is fully aligned with the standard care recommended for each respiratory diagnosis: chronic obstructive pulmonary disease (COPD) [1], asthma [2], and bronchiectasis [3].

Textbox 4. **Management of acute episodes.**

- In the event of a worsening of clinical conditions, the patient can contact the nurse case manager through the communication channel. The contact triggers the process of identifying a potential exacerbation, based on the patient’s reported symptoms (Modified Medical Research Council Dyspnoea Scale [mMRC] and COPD Assessment Test [CAT]), clinical assessment of the course, including oscillometry measurements. The nurse will be able to interact with the reference physician to decide on further steps needed for the diagnosis and management of the acute episode due to (1) lung-related events, (2) nonpulmonary dyspnea, or (3) dyspnea of mixed origin.
- Home-based management of the acute episode activates: (1) close follow-up of the patient, remote or face-to-face, by the nurse case manager, (2) daily patient’s self-administration of short questionnaires (Visual Analogic Scales [VAS]), as well as measurements of oscillometry and monitoring of physiological variables (Heart rate variability [HRV] and activity) with a wrist device, and (3) adjustment of the therapeutic plan in agreement with the reference physician.

This action plan will be maintained for 1 or 2 weeks depending upon the patient’s progress.

#### Roles of the Nurse Case Manager

As shown in Figure 2.1, the management of clinically stable candidates begins with a holistic assessment, followed by a personalization of the intensity and characteristics of the intervention. The primary aim of the hybrid care intervention is early identification and management of exacerbations at the community level, preventing progression to severe acute episodes requiring emergency room visits and/or unplanned hospitalizations. Beyond coordination and follow-up tasks, the nurse functions as the central clinical decision-making hub, integrating clinical information, patient-reported outcomes, and contextual factors to support anticipatory management and timely clinical responses.

Alignment between local clinical practices (standard operating procedures [SOP]) for each respiratory diagnosis and the characteristics of the hybrid care intervention, Textboxes 1–4 was achieved during PDSA 1 through regular meetings between the core research group and the clinical teams. Moreover, during PDSA 2, integration of information from electronic clinical records, patient questionnaires, nurse home visits, nurse-patient chat logs, and informal observations from nurses allowed personalization of the hybrid care intervention to fulfill individual patients’ needs, as reported in Table 1, Blocks 1 and 2.

#### Advanced Digital Support

As seen in Figure 2.2, the digital platform [12] acts as an enabling backbone of the intervention, supporting coordination, continuity of care, and timely clinical decision-making led by the nurse case manager. Briefly, the key features are: (1) multichannel communication between key professionals and patient, (2) shared care plans across health care tiers with an adaptive case management approach allowing flexibility in response to unexpected events and to specificities of different service providers, (3) patients’ data capture (short questionnaires, testing and monitoring physiological data), (4) cloud-based architecture to enable scalability, and (5) standard-based interoperability with existing corporate-specific health information systems. To be highlighted that the digital tools provided to the patient are customized by the nurse case manager according to the patient’s needs at each time, taking into consideration their digital literacy.

Technical developments to enhance acceptability and usability of different functionalities of the digital health platform (Table 1, Block 3) were active throughout the entire co-design process (PDSA-1 to PDSA-4). Decisions on the evolution of the Health Circuit platform were based on integration of key qualitative and quantitative inputs from the information sources indicated in Table 1.

#### Personalization of the Intervention

As seen in Figure 2.3, the health risk assessment component supports candidate profiling and the customization of care intensity according to individual needs and circumstances. Personalization of the intervention in the current research considered the following aspects: (1) severity of the respiratory disease, (2) comorbidity burden, (3) disability score, (4) socio-economic vulnerability, and (5) digital literacy. Findings from previous studies [23-36] provided key elements for addressing this component of the intervention through multilevel predictive models supporting clinical decision support, to be developed in future research.

During PDSA-3, integration of inputs from personalization of the intervention, achieved in the previous PDSA cycle, allowed defining the 6 stratification groups described in Table 3. Development and validation of computational modeling for clinical decision support is planned beyond the current co-design process.

**Table 3.** The six clinical groups defined in the study cohort.

Group	Main features	Hybrid care implications
Severe Asthma Program at HCB^a^ (n=48)		
A1 23 (47.92%)	Severe asthma, stage 5 [2], clinically stable without requiring interactions with the nurse	Provide access to the communication channel (app) without scheduling specific actions with the nurse.
A2 25 (52.08%)	Severe asthma, stage 5, exacerbations and/or other problems (mental disorders: mostly anxiety or depression, social frailty, …) trigger frequent interactions with the nurse	Target candidates for a hybrid care intervention. Management by an advanced care respiratory nurse. Specific needs were identified for A2 patients with severe asthma and obesity.
Community-based AISBE^b^ Program (n=135)		
C1 50 (37.04%)	Mild-moderate lung disease (ie, COPD^c^, GOLD^d^ I or II) with few symptoms and unproblematic comorbidities.	Not candidates for the intervention.
C2 18 (13.33%)	Moderate-severe lung disease, and/or rapid disease progress and/or history of severe exacerbations	Target candidate for the hybrid care intervention with shared care agreements with specialized care
C3 33 (24.44%)	Moderate-severe lung disease with problematic comorbidities and/or social frailty.	Target candidate requiring personalized medical (comorbidities) and social support [36].
C4 34 (25.19%)	Very severe lung disease (ie, COPD, GOLD IV) is often under home respiratory therapies.	Target candidate interacting closely with specialized care through an advanced care respiratory nurse.

a HCB: Hospital Clinic of Barcelona.

b AISBE: Integrated Health District of Barcelona-Esquerra.

c COPD: chronic obstructive pulmonary disease.

d GOLD: Global Initiative for Chronic Obstructive Lung Disease.

#### Characterization of the Exacerbations

As seen in Figure 2.4, during the exacerbations, patients perform daily, home-based patient data capture of oscillometry (lung function testing), HRV (autonomic regulation), and symptoms assessment (Visual-Analogic Scale) during the exacerbations (Appendix 4 in Multimedia Appendix 1). These home monitoring components are implemented as transient measures and are activated following the judgment of the nurse case manager. Regarding oscillometry, three major achievements are highlighted: (1) demonstration of applicability and acceptance of home-based patients’ self-measurements, (2) integration of oscillometry measurements in the digital platform for remote data evaluation by health professionals, and (3) assessment of concordance with forced spirometry and evaluation of measurement variability. At the end of PDSA-3, it was concluded that the approach is highly promising, contributing to objective assessment of acute episodes, but there is significant potential for improvement in terms of standardization of the technique [40].

Likewise, extensive clinical applicability of HRV measurements is limited by 2 traits of commercially available equipment: relatively high cost and poor accessibility to raw data.

The study generated relevant information on clinical applicability of household IAQ assessment with LCS [39] (Table 1, Block 4), but it was considered a topic to be addressed in future research.

### Clinical Management Pathways and Operational Features

Textboxes 1–4 summarize the clinical and operational characteristics of the hybrid care intervention at the end of the third PDSA cycle, detailing how the model articulates the roles of the main actors and the management procedures. These textboxes illustrates how the model is operationalized across care settings, distinguishing between the community-based AISBE program and the specialized Severe Asthma Unit at HCB.

Personalization of the hybrid care intervention (Table 3) was approached from three complementary perspectives: (1) clinical profiling of candidates, (2) contextual factors modulating care (eg, socio-economic, disability, living conditions); and (3) assessment of digital literacy.

Candidate profiling: baseline and midterm assessments, together with longitudinal monitoring and the contacts between the nurse case manager and participants over the first 18 months of follow-up, enabled the identification of six clinical groups: two within the severe asthma program (A1-A2) and 4 within the community-based AISBE program (C1-C4). These groups, and their corresponding hybrid care implications, were derived through an iterative profiling process conducted across the co-design cycles, integrating baseline characteristics, midterm assessment, and real-world observations over the follow-up period. The classification includes key dimensions: clinical severity of the respiratory disease, multimorbidity burden, functional status, socio-economic factors, and digital abilities, to inform the personalization of the hybrid care intervention. The characteristics of each group and their corresponding implications for hybrid care delivery are detailed in Table 3. Further information is displayed in Appendices 2‐3 in Multimedia Appendix 1.

These groups are intended to guide personalization of hybrid care rather than represent fixed clinical categories. Patient profiling and digital modality assignment represent complementary dimensions of personalization: the former defines care needs, while the latter reflects the level of digital support used to deliver the intervention.

Digital literacy: patients’ digital skills played a relevant role in the personalization of the hybrid care intervention. The level of digital skills directly influenced the type and intensity of digital support provided, ranging from phone-based management for patients with low or no digital skills or unwilling to use digital tools, to app-based communication and monitoring in those with higher or trainable digital capabilities. This allocation was dynamically adjusted by the nurse case manager according to patient progression and engagement. Briefly, 109 patients (59.6%) showed either high digital skills (n=51; 27.9%) or medium level scoring with significant improvements after training (n=58; 31.7%). The remaining patients presented significant limitations in the use of digital tools ranging from: (1) complete inability (n=16; 8.7%), or (2) poor digital skills (n=37; 20.2%), to (3) medium-level digital skills with no improvement after training (n=21; 11.5%) (Appendix 5 in Multimedia Appendix 1). Overall, 41 (22.4%) participants did not use the app, either due to clinical recommendation or personal preference. Improvements in digital literacy observed during follow-up appear to result from a combination of tailored training provided by the nurse case manager and progressive exposure to digital tools within the intervention, rather than from a single predefined training phase. At the end of PDSA-3, the distribution of the three different digital support modalities (M) was as follows: M1- phone-based follow-up (28% of the cohort), M2- app-based management with chat and patient-reported outcomes measures (PROMS; 21% of the cohort), and M3- app-based management including chat, PROMS, and HRV sensor (51% of the cohort). Allocation to each modality was personalized by the nurse case manager based on clinical characteristics, digital literacy, and patient engagement capacity and willingness.

Key lessons learned for scale-up of the hybrid-care intervention. The co-design process demonstrated applicability and stakeholders’ acceptance of the overall approach, contributing to the identification of some barriers partly limiting the digitally enabled management of the cohort. These findings provided emergent solutions for those limitations, as reported in Table 4. A major lesson learned was that further progress in the operationalization of the intervention must be made through its stepwise deployment in a real-world setting. Because of the direct relationships between patients and specialized professionals, the Severe Asthma program was selected as a priority scenario for deployment during the last PDSA cycle (PDSA-4).

Furthermore, several well-known consolidated frameworks guiding implementation processes were evaluated, aiming at fostering clinical adoption of the hybrid-care intervention. A pragmatic approach to the Integrated Theory-based Framework for Intervention Tailoring Strategies (ItFits-Toolkit) [41,42] was proposed to provide guidance. The selection of this implementation strategy was driven by two strategic priorities: (1) high potential for pragmatic application in the real-world setting covering the entire implementation cycle, and (2) plans for future use of these implementation tools at the regional level. This prospective framework is intended to bridge the gap between current operational results and systemic integration.

**Table 4.** Further refinements of the hybrid care intervention.

Intervention components	Expected improvements and outcomes
Digital support	Easy patient 2-factor authentication with biometrics (eg, biometric 2-factor authentication) to address login barriers Reduce the time required for synchronization of the wearable clinical device for monitoring of HRV^a^ Expand multimedia communication channel to other professionals Enhanced clinician’s dashboard Design of a novel voice-optimized interface, based on conversational agents powered by GenAI^b^.
Oscillometry and HRV	Develop and validate clear guidelines for home-based oscillometry use during exacerbations Create data-driven parameters for the management of moderate exacerbations at the community level. HRV: solving applicability issues for scaling-up of HRV data capture.
Foster sustainable adoption [41,42]	Refine personalized intervention protocols for the six identified clinical profiles. Expand engagement of all health care professionals Deployment of training programs for professionals Identification of KPIs facilitating program management Booklet guiding the implementation process Assessment of health care value generation [42]^c^
Risk assessment and clinical decision support	Identification of high-risk candidates Decision support tools for personalization of the intervention Parametrization of management of moderate exacerbations Proof of concept of the potential role of GenAI tools to support health professionals’ tasks

a HRV: heart rate variability.

b GenAI: generative artificial intelligence.

c Quintuple aim assessment: (1) health outcomes, (2) patient reported outcomes (PROMs) and patient reported experiences (PREMs), (3) health professionals’ experience, (4) direct costs evaluated with a cost–consequence analysis and (5) estimation of indirect costs to assess potential sources of inequity. See Appendices 4 and 6 in Multimedia Appendix 1 for detailed information.

## Discussion

The main study outcome from the reported co-design process was achievement of consensus across all key stakeholders at the end of PDSA-3 on two key aspects: (1) applicability of the hybrid care intervention in the real-world clinical setting, and (2) a well-defined strategy for its short-term deployment and sustainable site adoption. The co-design process operationalized the 3 core components of the hybrid care intervention: nurse-led management, personalized care, and advanced digital support.

Nurse-led management: the nurse case manager is central in the hybrid care intervention because of their role across several dimensions, namely: (1) comprehensive patient assessment and care personalization , (2) efficient integration across levels of health care and social support services, (3) community-based early management of exacerbations [43], and (4) dynamic adjustments of digital support, in terms of intensity and duration, to be aligned with patients’ transient or permanent needs, as well as to patients’ progress in terms of digital literacy. We acknowledge, however, that implementation of nurse-led management of hybrid care requires significant organizational reengineering of health care teams. Also, further work is required in terms of education and training of both community-based nurse case managers and specialized advanced care nurses within an integrated care scenario.

Personalized care: the identification of patients subgroupsin this study, based on a comprehensive characterization of the cases and the analysis of their requirements during the follow-up of the cohort was a first step toward personalized care. Future computational developments based on previous studies [23,24] should pave the way for elaboration and assessment of clinical decision support tools based on multilevel predictive modeling.

Advanced digital support: the architecture of the digital platform was perceived by stakeholders as a promising approach to address current limitations of digital support for collaborative work across health care tiers and service providers [4]. Digital literacy emerged as a critical determinant of patient engagement. From an implementation standpoint, these findings highlight a dual challenge: (1) ensuring that socially vulnerable patients are not excluded from digitally enabled care, and (2) allocating adequate resources for tailored training, simplified technological, and/or caregiver-mediated support. Our findings reinforce the strategic role of the nurse case manager, who not only delivers clinical care, but also facilitates effective adoption of digital tools.

At the end of the co-design process, the hybrid-care intervention was ready to be assessed in routine clinical practice within two well-differentiated ongoing clinical programs: (1) Severe Asthma Unit at HCB, and (2) Community-based management of high-risk chronic obstructive respiratory patients living in AISBE. The authors acknowledge the need to address challenges related to tailored implementation [41,42], and the assessment of health care value generation [44], before the intervention can be sustainably adopted and scaled up, as routine clinical care, aiming at optimizing the community-based management of acute episodes in these patients.

Two main limitations were identified in the execution of the study. First, the broad scope of the umbrella K-HealthinAir research project [16] required testing technologies for patients’ monitoring that showed different degrees of maturity, which added complexity during the first year (PDSA-1 and PDSA-2) with no impact on the final study results. The second, and most important factor, was the development of the co-design process within the frame of a research environment. This circumstance partially limited the role of the nurse in the management of acute episodes and delayed the assessment of the hybrid care intervention in a real-world scenario beyond the co-design process, after PDSA-4, as planned in the deployment strategy.

In conclusion, the co-design process enabled the operationalization of a hybrid care intervention integrating nurse-led management, personalization of care, and advanced digital support. Stakeholders reached consensus regarding its applicability and implementation strategy across 2 clinical programs. Future real-world implementation studies are required to assess its impact on health care use, patient outcomes, patient experience, and health care value generation.

## Supplementary material

10.2196/96600 Multimedia Appendix 1 Supplementary materials describing the co-design, implementation, and evaluation of the hybrid care intervention, including study context, baseline patient characteristics, disability and digital literacy assessments, exacerbation management, and predictive modeling with clinical decision support.

## Abbreviations

ACM: adaptive case management
AISBE: Integrated Health District of Barcelona-Esquerra
AMG: adjusted morbidity group
CHSS: Catalan Health Surveillance System
COPD: chronic obstructive pulmonary disease
FEV₁: forced expiratory volume in 1 second
FEV₁/FVC: ratio of FEV₁ to FVC
FVC: forced vital capacity
HCB: Hospital Clínic de Barcelona
HRV: heart rate variability
IAQ: indoor air quality
LCS: low-cost sensor
PDSA: plan-do-study-act
PROM: patient-reported outcomes measure
RCT: randomized controlled trial
SOP: standard operating procedure
WHODAS: World Health Organization Disability Assessment Schedule
