# Supplementary material for: A Hybrid Care Intervention for High-Risk Patients With Chronic Obstructive Respiratory Disorders: Mixed Methods Co-Design Study
Source: J Med Internet Res. 2026 Aug 18;28:e96600. doi: 10.2196/96600 (PMC13484947; doi:10.2196/96600)
Supplement: Supplementary file 1 [file jmir-v28-e96600-s001.docx]

**A Hybrid Care Intervention for High-Risk Patients With Chronic Obstructive Respiratory Disorders: Mixed Methods Co-Design Study**

*Alba Gómez-López et al.*

*-*

**Supplementary Material**

Contents

APPENDIX 1 – The context of the co-design process	2

APPENDIX 2 - Disability assessment across clinical groups	6

APPENDIX 3 - Baseline characteristics across clinical groups	7

APPENDIX 4 – Characterization of exacerbations	8

APPENDIX 5 - Digital literacy assessment	10

APPENDIX 6 – Predictive modelling and clinical decision support	11

References	12

# APPENDIX 1 – The context of the co-design process

The context and the study cohort

The context - The current mixed-methods research to co-design a hybrid care intervention for high-risk patients with chronic obstructive respiratory diseases was conducted under the umbrella of the EU project ‘Knowledge for improving indoor AIR quality and HEALTH (K-HEALTHinAIR, 2022–2026). The primary goal of this EU project was to enhance understanding regarding the impact of chemical and biological indoor air quality (IAQ) on human health. Moreover, the project aimed to devise cost-effective methods for more precise monitoring and improvement of IAQ.

The leading team of the Barcelona Pilot in K-HEALTHinAIR (AGL, ICF, RGC and JRT) pertains to the Integrated Care and Systems Medicine group (INCASyM) from IDIBAPS-Hospital Clinic de Barcelona, with expertise on digital health transformation to support integrated care management of patients with chronic conditions. The group has a long-track record in three areas: (1) design, assessment and implementation of innovative integrated care services, (2) multilevel predictive modelling of health risk assessment, and (3) digital technologies. The background work supporting the current research was reported in three recent PhD thesis [1-3].

INCASyM is part of a robust collaborative network of multidisciplinary professionals supporting the evolving process of an innovative integrated care service from its initial evaluation of efficacy to assessment of effectiveness and scale-up at regional level. As described in the main manuscript, such network of multidisciplinary professionals has been supporting the co-design process since its inception.

The core research team including clinical professionals (AGL, JRT), one data scientist (RGC) and one digital health specialist (ICF) coordinated the interactions, throughout the two-year follow-up, with the patients included in the cohort (AGL), and with the clinical teams in primary care (NSR, MSC, MJG), pneumologists (MPL, JSY, NSP, EAN) and with two engineers and one physicist experts in the different technologies tested in the study (AAP, JFD, RFV), as described in the main manuscript. Moreover, close to the end of PDSA-3, professionals from the Digitalization for the Sustainability of the Healthcare System (DS3 group) pertaining to the single regional public payer in Catalonia (CatSalut) joined the research program. All these people, except the technical team (AAP, JFD, RFV), participated in the final focus group session described in the main manuscript.

The Study Cohort – The candidates targeted for the study cohort were patients with chronic obstructive respiratory disorders: COPD, asthma or bronchiectasis, showing severe respiratory disease and/or co-morbid conditions leading to a health high-risk profile. To this end, patients’ enrolment focused on two programs: (1) Community-based management in AISBE, and (2) Severe Asthma program at HCB. The patients included in the community-based program were allocated close to the tip, above percentile 80, of the population-based health risk assessment pyramid from Catalonia, using Adjusted Morbidity Groups (AMG) [4, 5] score, as morbidity quantifier. The severe asthma group were highly selected patients managed in the outpatient clinic of the tertiary hospital. The mean follow-up of the study cohort was 558.5 days (SD 164.6), with a median of 602 days.

KHEALTHinAIR [6] aimed at household indoor air quality (IAQ) monitoring using low-cost sensors to explore the relationships between IAQ and health status. The setting of the EU project provided a unique scenario to analyse the potential of a hybrid care intervention for the management of these patients during clinical stability, as well as during acute episodes. The underlying hypothesis was that hybrid care could facilitate early detection and optimal management of community-based exacerbations preventing emergency room visits and unplanned hospitalizations. However, the complexities of the approach required a co-design process to achieve a hybrid care intervention with high applicability and scalability.

The co-design process

The co-design process was structured into four sequential Plan–Do–Study–Act (PDSA) cycles conducted over a two-year period, from January 2024 to December 2025, as outlined in the study protocol [7]. The originally planned six-month duration of each PDSA cycle was slightly modified, with a two-month extension of PDSA-3 to enable full maturation of the core characteristics of the hybrid care intervention. The positive acceptance of the approach by the various stakeholder groups prompted the preparation of this report and the initiation of the fourth PDSA cycle, which runs from September 2025 to February 2026 (**Table S1 and Table 4**). Subsequent steps will include the tailored implementation of the hybrid-care intervention in real-world settings beyond the current study cohort, as well as an evaluation of its healthcare value generation.

During the first three PDSA cycles, the four members of the core research team: nurse case manager (AGL), a respiratory specialist (JRT), a data scientist (RGC), and a digital health specialist (ICF) jointly refined the different components of the hybrid care intervention (**Figure 2**) through continuous interaction with participants, patients and caregivers, in the study cohort (n=205). The hybrid care intervention was implemented across two clinical programs: (i) integrated care for AISBE (asthma, COPD, and bronchiectasis), jointly led by respiratory specialists (NSP) and primary care professionals (NSR, MSC, MJG), representing 74% of the study cohort; and (ii) the Severe Asthma Unit at HCB, led by EAN, encompassing the remaining 26% of participants (**Figure 1**). These clinical leaders provided sustained guidance and feedback throughout the co-design process. In addition, partners from the EU project *K-HealthinAir* (JFD and AAP) contributed continuous input on technical aspects of household indoor air quality (IAQ) monitoring, while RFV offered expertise on Oscillometry-related topics. Several workshops and meetings with the collaborators mentioned above were held through the co-design process.

At the conclusion of PDSA-3, an early draft of the main manuscript was shared with the participants in a two-hour focus group session held in August 2025. The session addressed three key questions: (i) the structure and components of the hybrid care intervention; (ii) barriers and facilitators for its tailored implementation in real-world settings; and (iii) the clarity and coherence of the draft manuscript. The focus group provided valuable insights, and all participants contributed to refining the final version of this manuscript.

The fourteen focus group participants represented a diverse range of professional profiles, including respiratory specialists (EAN, MPL, NSP, JSY, JRT), primary care professionals (NSR, MJG, MSC, AGL), digital health specialists and data scientists (ICF, XMV, RGC), and healthcare managers (GCS, AJR).

**TABLE S1 – Adapted co-design protocol for the enhanced management of multimorbid patients with COPD or severe asthma: role of IAQ [7]**.

| **Analysis of the relationships between IAQ and health status in the patients’ cohort** **(n=205) within the EU project K-Health in Air** (https://k-healthinair.eu/), **and tailored implementation plans for sustainable adoption of the hybrid care intervention in real-world settings** | |
| --- | --- |
| **# Cycle** | **Objectives** |
| **PDSA-1**  *(January – June 2024)* | Analysis of feasibility and clinical applicability of the components of the hybrid care intervention as planned in the seminal protocol [7]: (1) roles of the nurse leading the hybrid care intervention in the two clinical programs, (2) personalization of care with clinical decision support tools (predictive modelling), (3) Advanced digital support, and (4) household indoor air quality (IAQ) monitoring using low-cost sensors (LCS). |
| **PDSA-2**  *(July – December 2024)* | Finetuning of the core components of the intervention following the requirements identified in PDSA-1. During the co-design process, the nurse case manager had continuous interactions with patients and caregivers capturing reported outcomes and experiences. Reporting on preliminary outcomes of IAQ monitoring using LCS was released at the end of this PDSA cycle (see main manuscript). |
| **PDSA-3 (*)**  *(January – August 2025)* | Completion of finetuning of the hybrid care intervention for clinical care. Operationalization of the core components of the interventions. Applicability and acceptability assessed by the focus group session. |
| **PDSA-4**  *(September 2025 – February 2026)* | (1) Progress towards clinical adoption of the basic version of the hybrid care intervention, beyond the study cohort, prioritizing actions in the Severe Asthma Unit. (2) Evolutions of the components of the intervention (see **Table 4**, **Appendices 4** and **6**). (3) Preparation of the implementation tailoring program, as well as assessment of healthcare value generation, addressing the high-risk patients included in the AISBE program, mainly COPD patients. |

*^(*)^ Timeframe of the current report, from PDSA-1 to PDSA-3; IAQ: Indoor Air Quality; LCS: Low-Cost Sensors; Symptoms:  mMRC/CAT questionnaires; HR: Heart Rate; HRV: Heart Rate Variability; PDSA: six-month co-design cycles using Plan-Do-Study-Act [8] methodology.*

Household IAQ monitoring with Low-cost sensors (LCS)

The results of the household IAQ monitoring **(Figure 2.5)** of the study cohort were reported in [9]. Briefly, IAQ was profiled prospectively over two-month period registering concentrations of CO₂, PM₂.₅ and formaldehyde every 10 min. For each pollutant, dwellings were classified as Good, Moderate or Unhealthy according to the Global Open Air Quality Standards thresholds (GO-AQS) [10].

Ninety-two homes (51.7%) had at least one pollutant in an at-risk category. The burden was mostly generated by PM_2.5_: 40.1% of dwellings were classified as at risk (32.8% Moderate; 7.3% Unhealthy). Formaldehyde exceeded the low-risk threshold in 22 homes (12.4%). Tobacco smoking, either active or passive, was significantly associated with PM₂.₅ levels (p<0.001).

It was concluded that household PM_2.5_ driven pollution is prevalent and strongly linked to indoor smoking. LCS are well-suited for household IAQ risk screening in selected chronic respiratory patients, but continuous long-term monitoring is not efficient. Household IAQ assessment in the clinical scenario should be reserved for selected cases in which environmental exposure was clinically suspected to contribute to symptom persistence or exacerbation risk.

*Validation and concordance of respiratory diagnoses*

As described in the Methods section, the co-design process assessed the validation of the respiratory diagnosis. Briefly, 62% of the patients (n=114) showed coincidence between the respiratory diagnosis validated by the research team and the registry information. The regional registry (CHSS) contained two respiratory diagnoses in 30% of the cases (n=56), three respiratory diagnoses in 7% (n=12), and none in 1% (n=3). No misclassifications were observed in the Severe Asthma Clinic program, whereas the AISBE program showed misclassifications of diagnoses in asthma (n=25) and bronchiectasis (n=40).

# APPENDIX 2 - Disability assessment across clinical groups

***Figure S1 – Disability assessment*** *using the WHODAS 12.0 [11] questionnaire. Boxplots of the assessment of disability for the six dimensions of the questionnaire. The first boxplot corresponds to the entire study cohort, whereas the subsequent six boxplots correspond to the results obtained for each of the clinical groups. Disability domains: Cognition (understanding/communicating), Mobility (moving/getting around), Self-care (hygiene, dressing, eating, staying alone), Getting along (interacting with others), Life activities (household/work), and Participation (community participation and societal involvement).*

**Clinical Groups Overview**

**A1:** clinically stable severe asthma (step 5) , infrequent nurse interactions.
**A2:** unstable severe asthma with psychosocial comorbidity (e.g., anxiety/depression, social frailty); frequent nurse interactions.
**C1:** COPD/bronchiectasis with mild–moderate impairment, few symptoms, non-problematic multimorbidity.
**C2:** moderate–severe respiratory disease and/or rapid progression and/or prior severe exacerbations.
**C3:** moderate–severe respiratory disease plus problematic multimorbidity and/or social frailty.
**C4:** very severe disease (often COPD GOLD IV), frequently on home respiratory therapies.

# APPENDIX 3 - Baseline characteristics across clinical groups

***Figure S2. Stacked distributions of key baseline characteristics across clinical groups.*** *Stacked bar charts showing the relative distribution of the six clinical groups (A1–A2: Severe Asthma program; C1–C4: AISBE community program) across baseline variables. Panels depict age, sex, AMG [4, 5] risk strata, WHO-5 well-being [12], PROMIS-10 Global Mental and Physical Health [13], airflow-limitation, Dyspnoea[14], and WHODAS-12 [11] disability severity. Bars are normalized to 100% within each category; numbers inside bars are counts; and numbers above facet strips indicates the number of observations for that level. * AMG: Adjusted Morbidity Groups score. mMRC: Modified Medical Research Council Dyspnoea Scale; FEV1: Forced Expiratory Volume in 1 second.*

# APPENDIX 4 – Characterization of exacerbations

Early objective characterization, and management, of community-based, exacerbations in high-risk candidates, following accepted clinical guidelines[15-17], is one of the core objectives of the hybrid care intervention. **Figure 2**.4, and the corresponding text of the main manuscript, describe the main traits of the management of acute episodes.

Briefly, daily patients’ data capture during exacerbations, involves: (1) Symptoms through a visual analogic scale (VAS), (2) home-based self-measurement of oscillometry (respiratory mechanics), and (3) overnight monitoring of heart rate variability, HRV, reflecting the balance between adrenergic and vagal autonomic regulatory systems. The preliminary results obtained at the end of PDSA-3 are promising, but well-identified items are requiring attention during PDSA-4, as follows:

**Symptoms (VAS)**: Three types of questionnaires for daily assessment have been tested through the co-design process: mMRC [14], modified COPD Exacerbation Recognition Tool (CERT) [18] and EXAcerbations of Chronic Pulmonary Disease Tool (EXACT) [19]. All of them are applicable and useful but show significant intraindividual variability. The final decision will be taken at the end of PDSA-4. Likely, in favour of the simplest option.

**Oscillometry [20-23]**: Major achievements are reported in the main manuscript. At the end of PDSA-3, it is concluded that the approach is highly promising, but there is a significant potential for improvement. Further refinements in the standardization of the calculations consisting of analysis of the expiratory phase of the breathing cycle and reformulation of key variables with a pragmatic approach are required. The ultimate twofold objective is: (1) reduction of the minimal clinically important difference (MCID), and (2) simplification of the interpretation of the oscillometry outcomes for routine clinical use. Ongoing analysis of oscillometry data collected during the first eighteen-month follow-up of the study cohort should allow to properly address these two aspects within PDSA-4. A specific report on oscillometry is being prepared.

**HRV**: Main outcomes of the co-design process are: (1) key learnings on data capture in terms of applicability and patients’ acceptability of sensors (wrist sensors) and standardization of measurements (mean overnight registries) [24], (2) despite an overnight intraindividual variability of HRV, daily mean overnight values seem to reflect consistent changes of the autonomic regulatory system during exacerbations, as shown in **Figure S3** depicting the evolution of HRV during a viral respiratory infection in one patient.

***Figure S3 - Evolution of mean overnight heart rate variability (HRV, ms) across consecutive days in a single individual during a viral respiratory infection.*** Mean overnight HRV shows a slight anticipatory decline preceding the clinical exacerbation, followed by a pronounced reduction during the first 24 hours of the acute phase. Subsequently, HRV exhibits a gradual and progressive recovery over the following days, approaching baseline levels.

The main pending challenge regarding HRV is the identification of a commercial wrist sensor certified as a medical device, with a reasonable cost facilitating cost-efficiency of the measurements. The interoperability of such sensor into the digital platform will be required but is a relatively minor issue.

During PDSA-4, achievement of an operational integration of the three measurements. VAS, Oscillometry and HRV in a simple and cost-efficient manner constitutes one of the central challenges of the period. Moreover, a proper representation of the information capture to facilitate clinical decision making will be required, as briefly analysed in **Appendix 6**. Transient monitoring during acute episodes, based on daily home self-administered oscillometry, HRV, and symptom tracking, may be incorporated as an on-demand module to support early identification and objective characterization of exacerbation onset and recovery.

As indicated in **Table 2,** the optimization of the approach requires patients’ proactivity and empowerment for self-management, ensuring communication with the nurse in an early phase of an acute episode. For the community-based program (i.e. AISBE), the nurse case manager is a trained professional for delivering and tailoring the intervention according to each patient’s profile. The reference primary care physician supervises the activity of the nurse, who also has easy access to specialized care (physicians and/or advanced care nurses) if needed. For specialized care programs (i.e. Severe Asthma Unit), the health professional leading the program has the profile of an advanced care nurse integrated into the specialized asthma team.

# APPENDIX 5 - Digital literacy assessment

***Figure S4: Digital literacy.*** *Distribution of the patients (dots) according to their baseline digital literacy scoring (X-axis) against the requirements in terms of support and training (Y-axis) resulting from the twelve questions evaluated in the survey.* Categorical responses were binarized, and a Principal Component Analysis (PCA) was applied to identify underlying patterns. *The colors indicate the six groups resulting from the digital literacy scoring subjectively assessed by the nurse case manager: i) high digital skills (garnet); ii) medium scoring with improvement after training (dark blue), and iii) without improvement after training (dark green); iv) low digital skills (lighter green); v) no skills after using the digital tools (light green); and vi) digitally excluded by the nurse, never used the digital tools available in the study (yellow. The discontinuous lines indicate the area including 95% of the individuals in each of the six groups identified). The boxplots of digital skills (X-axis) and requirements of support (Y-axis) for the five groups using the digital tools are also depicted in the figure.*

**TABLE S2 – Digital literacy by clinical program**

| **Digital literacy** | **All patients (n=183)** | **Patients followed by Primary Care (n=135)** | **Patients followed by the Severe Asthma Unit (n=48)** | **p- value** |
| --- | --- | --- | --- | --- |
| None; n (%) | 16 (8.74%) | 15 (11.11%) | 1 (2.08%) | .012 |
| Low; n (%) | 37 (20.22%) | 30 (22.22%) | 7 (14.58%) |  |
| Medium (no improvement); n (%) | 21 (11.48%) | 18 (13.33%) | 3 (6.25%) |  |
| Medium (can improve); n (%) | 58 (31.69%) | 43 (31.85%) | 15 (31.25%) |  |
| High; n (%) | 51 (27.87%) | 29 (21.48%) | 22 (45.83%) |  |

The digital support to the hybrid care intervention is provided by the Health Circuit [25] platform and was refined during the co-design process. The two most critical functions limiting the use of digital tools were: i) login to the App, and ii) synchronization of the wrist sensor. In all cases, the characteristics, and duration, of the digital support were customized according to the individual level of digital literacy and the patient’s requirements, assessed by the nurse.

# APPENDIX 6 – Predictive modelling and clinical decision support

The two-year follow-up study of a cohort of 205 high-risk respiratory patients with obstructive disorders shows that the hybrid care intervention is applicable and has a very highly level of acceptance among all stakeholders enabling personalized, scalable care while showing potential for healthcare value generation. In this context, advanced data analytics techniques can provide substantial added value in terms of knowledge generation and efficiencies to the clinical approach. To this end, the codesign process identified the following three main areas of action:

**Health Risk Assessment (HRA) component** represents the core analytical element of the hybrid care model, supporting candidate’s profiling and the stratification of care intensity according to individual needs and circumstances. It incorporates Machine Learning–based risk assessment models to identify high-risk patients that would benefit the most from personalization of the intervention, following the recommendations generated in [1, 26, 27] . The central aim is to refine, and enrich, the personalization of the hybrid care intervention in the six clinical groups identified in the study cohort. The knowledge acquired during the co-design process will be applied to the clinical scenario at the end of the present follow-up study.

**Characterization of the exacerbations**. The main manuscript, and **Appendix 4**, summarize the current challenges to be solved within PDSA-4. Data analytics of the patient’s information captured during the exacerbations (VAS, oscillometry and HRV) is expected to provide highly valuable tools for clinical management of the acute episodes, to be evaluated in real-world settings beyond the current study.

**Use of AI agents for** **the capture and synthesis of clinical information**. Within PDSA-4, mid-February 2026, a Datathon challenge aims to deliver a proof of concept for a conversational agent that seamlessly integrates into existing clinical workflows. The agent collects structured symptom information via natural voice interactions with patients or caregivers, automatically integrates home-based functional testing and continuous monitoring data, and produces a concise, structured daily clinical report to support nurse-led case management and physician decision-making. The emphasis is on augmenting clinical capacity, not replacing human oversight.

# References

1.	Gonzalez-Colom R (2024). Predictive Modelling for Personalised Multimorbidity Management.: University of Barcelona.https://hdl.handle.net/10803/691632

2.	Herranz C (2023). An Adaptative Case Management Approach To Prevent Unplanned Hospital Admissions in a Care Continuum Scenario.: University of Barcelona.https://hdl.handle.net/2445/215620

3.	Baltaxe E (2023). Evaluation and Digital Transformation Integrated Care Services: University of Barcelona.https://hdl.handle.net/10803/692258

4.	Monterde D ET AL. Adjusted morbidity groups: A new multiple morbidity measurement of use in Primary Care. *Aten Primaria*. 2016;48(10):674–82. PMC6879171. doi: 10.1016/j.aprim.2016.06.003

5.	Cerezo Cerezo, José & Arias López, Carmen (2018). Population stratification: a fundamental instrument used for population health management in Spain: good practice brief. World Health Organization. Regional Office for Europe. https://iris.who.int/handle/10665/345586.

6.	K-Health in Air (2022 - 2026). Knowledge for Improving Indoor Air Quality and Health (K-Health in Air). Available from: https://k-healthinair.eu/.

7.	Gomez-Lopez A ET AL. Protocol for the enhanced management of multimorbid patients with COPD and severe asthma: role of indoor air quality. *BMJ Open Respir Res*. 2025;12(1). PMC11752068. doi: 10.1136/bmjresp-2024-002589

8.	Christoff P. Running PDSA cycles. *Curr Probl Pediatr Adolesc Health Care*. 2018;48(8):198–201. doi: 10.1016/j.cppeds.2018.08.006

9.	González-Colom R, Gómez-López A, Aguado A, et al. Should household air quality monitoring be considered in selected patients with asthma and COPD? BMJ Open Respir Res. Jul 1, 2026;13(1):e003899. [doi: 10.1136/bmjresp-2025-003899] [Medline: 42386347]

10.	Global Open Air Quality Standards (GO AQS), 2025, Global Open Indoor Air Quality Standards: A Unified Framework, November 2025, Version 1.0; ISBN: 9798274916158.

11.	Thomas CC ET AL. The 12-item WHO Disability Assessment Schedule II as an outcome measure for treatment of common mental disorders. *Glob Ment Health (Camb)*. 2016;3:e14. PMC5314748. doi: 10.1017/gmh.2016.7

12.	Topp CW ET AL. The WHO-5 Well-Being Index: a systematic review of the literature. *Psychother Psychosom*. 2015;84(3):167–76. doi: 10.1159/000376585

13.	Riggs JS ET AL. PROMIS Global Health: Feasibility in home health. *Qual Life Res*. 2021;30(9):2551–61. doi: 10.1007/s11136-021-02845-x

14.	Stoller JK ET AL. Further specification and evaluation of a new clinical index for dyspnea. *Am Rev Respir Dis*. 1986;134(6):1129–34. doi: 10.1164/arrd.1986.134.5.1129

15.	Agusti A ET AL. Global Initiative for Chronic Obstructive Lung Disease 2023 Report: GOLD Executive Summary. *Am J Respir Crit Care Med*. 2023;207(7):819–37. PMC10111975. doi: 10.1164/rccm.202301-0106PP

16.	2025 GINA strategy report: global strategy for asthma management and prevention. Global Initiative for Asthma (GINA); 2025. URL: <https://ginasthma.org/2025-gina-strategy-report/>

17.	Polverino E ET AL. European Respiratory Society guidelines for the management of adult bronchiectasis. *Eur Respir J*. 2017;50(3). doi: 10.1183/13993003.00629-2017

18.	Jones PW ET AL. The Development of a COPD Exacerbation Recognition Tool (CERT) to Help Patients Recognize When to Seek Medical Advice. *Int J Chron Obstruct Pulmon Dis*. 2022;17:213–22. PMC8789323. doi: 10.2147/COPD.S337644

19.	Leidy NK ET AL. Development of the EXAcerbations of Chronic Obstructive Pulmonary Disease Tool (EXACT): a patient-reported outcome (PRO) measure. *Value Health*. 2010;13(8):965–75. doi: 10.1111/j.1524-4733.2010.00772.x

20.	King GG ET AL. Technical standards for respiratory oscillometry. *Eur Respir J*. 2020;55(2). doi: 10.1183/13993003.00753-2019

21.	Veneroni C ET AL. Diagnostic Potential of Oscillometry: A Population-based Approach. *Am J Respir Crit Care Med*. 2024;209(4):444–53. PMC10878374. doi: 10.1164/rccm.202306-0975OC

22.	European Respiratory Society. Respiratory Physiology: New Knowledge, Better Diagnosis. Dellacà R, Aliverti A, editors. European Respiratory Monograph; no. 107. Sheffield: European Respiratory Society; 2025. 310 p. ISBN: 978-1-84984-188-7.

23.	Simpson S, Irvin CG, King GG, Veneroni C, editors. Oscillometry: clinical significance and applications. European Respiratory Society; n.d.

24.	Shaffer F, Ginsberg JP. An Overview of Heart Rate Variability Metrics and Norms. *Front Public Health*. 2017;5:258. PMC5624990. doi: 10.3389/fpubh.2017.00258

25.	Herranz C ET AL. A Practice-Proven Adaptive Case Management Approach for Innovative Health Care Services (Health Circuit): Cluster Randomized Clinical Pilot and Descriptive Observational Study. *J Med Internet Res*. 2023;25:e47672. PMC10337458. doi: 10.2196/47672

26.	González-Colom R ET AL. Prevention of Unplanned Hospital Admissions in Multimorbid Patients Using Computational Modeling: Observational Retrospective Cohort Study. *J Med Internet Res*. 2023;25:e40846. PMC9982720. doi: 10.2196/40846

27.	González-Colom R CI, Piera-Jiménez J, Roca J. Multilevel Modelling with AI: The Synergy-COPD Endeavour BT - Personalized Medicine Meets Artificial Intelligence: Beyond “Hype”, Towards the Metaverse. In: Cesario A DOM, Auffray C, Scambia G, editors, editor. Springer International Publishing;10.1007/978-3-031-32614-1_102023. p. 131–44.
